# KRT20, KRT5, ESR1 and ERBB2 Expression Can Predict Pathologic Outcome in Patients Undergoing Neoadjuvant Chemotherapy and Radical Cystectomy for Muscle-Invasive Bladder Cancer

**DOI:** 10.3390/jpm11060473

**Published:** 2021-05-26

**Authors:** Hendrik Jütte, Moritz Reike, Ralph M. Wirtz, Maximilian Kriegmair, Philipp Erben, Karl Tully, Veronika Weyerer, Markus Eckstein, Arndt Hartmann, Sebastian Eidt, Felix Wezel, Christian Bolenz, Andrea Tannapfel, Joachim Noldus, Florian Roghmann

**Affiliations:** 1 Institute of Pathology, Ruhr-University Bochum, 44789 Bochum, Germany; andrea.tannapfel@pathologie-bochum.de; 2 Department of Urology, Marien Hospital, Ruhr-University Bochum, 44625 Herne, Germany; moritz-johannes.reike@elisabethgruppe.de (M.R.); karl.tully@elisabethgruppe.de (K.T.); joachim.noldus@elisabethgruppe.de (J.N.); florian.roghmann@elisabethgruppe.de (F.R.); 3 Stratifyer Molecular Pathology GmbH, 50935 Cologne, Germany; ralph.wirtz@stratifyer.de; 4 Department of Urology and Urosurgery, Medical Faculty Mannheim, University of Heidelberg, 68167 Mannheim, Germany; maximilian.kriegmair@umm.de (M.K.); philipp.erben@umm.de (P.E.); 5 Institute of Pathology, University Hospital Erlangen, Friedrich-Alexander-Universität Erlangen-Nürnberg, 91054 Erlangen, Germany; veronika.weyerer@uk-erlangen.de (V.W.); markus.eckstein@uk-erlangen.de (M.E.); Arndt.Hartmann@uk-erlangen.de (A.H.); 6 Institute of Pathology, St. Elisabeth Hospital, 50935 Cologne, Germany; sebastian.eidt@gmx.de; 7 Department of Urology, University Hospital Ulm, University of Ulm, 89081 Ulm, Germany; felix.wezel@uniklinik-ulm.de (F.W.); Christian.Bolenz@uniklinik-ulm.de (C.B.)

**Keywords:** bladder cancer, chemotherapy, muscle invasive, risk stratification, subtype

## Abstract

Patients with muscle-invasive bladder cancer (MIBC) that underwent neoadjuvant chemotherapy (NAC) prior to radical cystectomy (RC) show improved overall survival, especially those with pathological complete response (pCR). The response to NAC according to molecular subtypes has been discussed. Molecular targets such as estrogen receptor (ESR1) and human epidermal growth factor receptor 2 (ERBB2) play an important role in breast cancer management and have also been associated with urothelial bladder cancer. Hence, the association of Keratin 20 (KRT20) Keratin 5 (KRT5), ESR1, and ERBB2 mRNA expression in MIBC at transurethral resection (TUR-BT) with pCR after NAC was analyzed retrospectively. Formalin-fixed paraffin-embedded tumour tissue samples from TUR-BT of 54 patients (42 males, 12 females, median age of 64) with MIBC were analyzed for KRT20, KRT5, ESR1, and ERBB2 mRNA expression. After NAC, RC was performed, and the specimens were evaluated for pCR. Statistical analyses comprised nonparametric and chi^2^ testing, partition models, and Spearman correlation analyses. After NAC, 22 out of 54 patients (40.7%) had pCR. Tumours with an elevated expression of markers associated with luminal differentiation (KRT20, ERBB2, ESR1) were associated with a higher chance of pCR (55% vs. 15.8%, *p* = 0.009). Elevated ERBB2 expression was positively correlated with luminal expression features such as KRT20, and negatively with basal characteristics such as KRT5. Patients with MIBC showing a high expression of ERBB2, ESR1, or KRT20 have a significantly higher chance of pCR following NAC. These findings might improve patient selection for NAC in MIBC.

## 1. Introduction

Muscle-invasive bladder cancer (MIBC) is an aggressive disease with a five-year survival rate of less than 15% if untreated [1]. Half of the patients progress to metastatic disease within two years of diagnosis [1]. The gold standard for MIBC treatment is radical cystectomy (RC) with bilateral pelvic lymph node dissection [2]. Administration of platinum-based neoadjuvant chemotherapy (NAC) before surgical resection has been demonstrated to improve the overall survival rates and the pathological complete response (pCR) and to decrease residual disease in patients with cT2-T4a-cN0cM0 MIBC [3,4]. Both the European and American [5,6] guidelines therefore recommend a platinum-based therapy for these patients. Nonetheless, patients may be ineligible [7] for either chemotherapy or RC, and selection of the most appropriate therapy depends on a staging system that may not accurately determine the eligibility of a patient for a specific treatment plan [8,9]. Thus, a more personalised approach to tumour subtyping and subsequent therapy management is warranted [10].

Genetic profiling has contributed to a more precise distinction between molecular subtypes of MIBC and allows for an individualised risk assessment and the identification of potential therapeutic target genes [11,12,13,14]. These molecular subtypes show a distinct clinical behaviour and respond differently to targeted therapies. The expression levels of biomarkers such as hormone receptors and tyrosine kinase receptors have been shown to directly correlate with the response to NAC and the outcome in breast cancer patients [15]. Denkert et al. demonstrated the clinical value of oestrogen receptor alpha (ESR1) and human epidermal growth factor receptor 2 (ERBB2) mRNA expression analysis in predicting pCR [15,16].

In bladder cancer, gene expression patterns have been established to distinguish between low-grade and high-grade cancers, between primary and secondary tumours, and between basal and luminal subtypes. Yet, studies assessing such patterns to categorise subtypes within high-grade MIBC are comparatively scarce [13,17,18]. Similar to breast cancer, MIBC is classified into luminal and basal subtypes based on the expression of biomarkers such as cytokeratins and cell surface proteins [13]. Moreover, ESR1 and ERBB2 are expressed in MIBC and may predict response to therapy [19]. However, it remains a matter of debate whether their expression patterns are comparable to those in breast tumours. Mostly, MIBC originate from basal or luminal cells. Nonetheless, the classification of bladder cancers still relies on the standardized histopathological assessment of the tumour tissue without molecular subtyping and, hence, no differentiation between luminal and basal subtypes [13]. Categorisation of MIBC subtypes based on mRNA expression levels of such biomarkers might be directly indicative of the clinical outcome of MIBC patients [13,20].

In the present work, we therefore analysed the association of basal and luminal mRNA expression patterns such as Keratin 20 (KRT 20), Keratin 5 (KRT5), as well as ESR1 and ERBB2 in patients with MIBC at transurethral resection (TUR-BT) with pCR at RC after platinum-based chemotherapy.

## 2. Materials and Methods

### 2.1. Study Population

**Figure 1:**
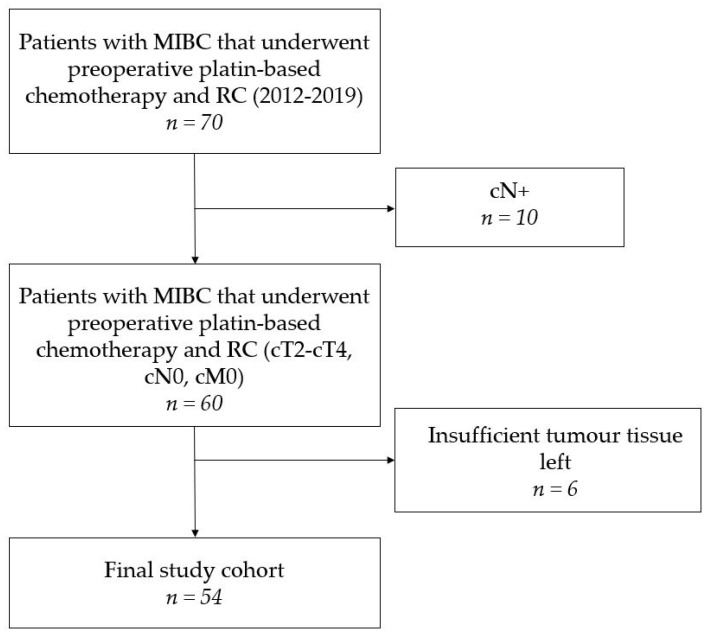
Flowchart of the study cohort. (MIBC: muscle-invasive bladder cancer, NAC: neoadjuvant chemotherapy, RC: radical cystectomy).

A cohort of 70 consecutive patients with MIBC that underwent preoperative chemotherapy and RC at the Department of Urology of the Ruhr-University Bochum (Marien Hospital, Herne, Germany) between January 2012 and November 2019 was identified (Figure 1). Initial clinical staging consisted of bimanual palpation, consecutive TUR-BT as well as baseline computed tomography (CT) of thorax, abdomen and pelvis. Inclusion criteria were urothelial carcinomas with clinical stages cT2–cT4, cN0 and cM0, while only a partial squamous differentiation (less than 10%) was accepted. Exclusion criteria were other histological variants of urothelial carcinoma, non-urothelial entities such as small cell neuroendocrine carcinoma, a glomerular filtration rate (GFR) of <50 mL/min, Karnofsky index below 60% (ECOG > 1), hearing loss, and polyneuropathy [20]. Therefore, 10 patients with radiologically confirmed lymph node metastases were excluded from further analyses (Figure 1). Formalin fixed paraffin embedded (FFPE) tumour tissue samples from TUR and RC of the remaining 60 patients was obtained. Insufficient tissue led to the exclusion of another six patients resulting in a final study population of 54 patients (pCR cohort, Figure 1). The majority of the patients was male 42/54 (78%) and had a median age of 64 (IQR 59–69) with histologically confirmed MIBC [7]. Median Body Mass Index (BMI) was 26.49 (IQR 24.4–29.72). The American Society of Anesthesiologists physical status classification system (ASA-score) was collected before RC. In our cohort, 7 patients were diagnosed with a malignant disease before inclusion. No cisplatin-based chemotherapy has been applied to patients in our cohort. Patients were treated with NAC according to the well-established regimen of gemcitabine (1250 mg/m^2^) on d1 and d8 and Cisplatin (70 mg/m^2^) on d1 every 3 weeks, with a median of 3 (IQR 2–3) cycles. According to national guideline recommendations, an interim staging consisting of a CT scan (thorax, abdomen, pelvis) was conducted following the completion of the 2 cycles. In case of tumour progression at interim staging or dose limiting toxicity, RC was performed immediately. Patients with a GFR of 50–60 mL/min received a splitting dose regimen with gemcitabine (1250 mg/m^2^) on d1 and cisplatin (35 mg/m^2^) on d1 and d2 [21]. Baseline characteristics of the study population are displayed in Table 1. All patients gave informed consent and study approval was obtained from the local ethics committees (4047-11). 

### 2.2. Pathologic Assessment

The haematoxylin-eosin (HE) stained tissue sections taken from the transurethral resection (TUR) prior to NAC and from the surgical specimen thereafter were evaluated and classified according to the current TNM-classification of the UICC (2017) by three experienced uro-pathologists (AH, SE, HJ). In the case of pCR, the whole tumour bed was processed in depth and used as comparator. In this study, pCR was defined in such a way that no vital tumour was detectable after NAC in the cystectomy and lymphadenectomy specimen.

### 2.3. Molecular Pathology 

RNA was isolated from FFPE tissue using 10 µm sections. These were processed automatically by a bead-based extraction method as described (XTRACT kit; STRATIFYER Molecular Pathology GmbH, Cologne, Germany). RNA was eluted with 100 µL elution buffer and then analysed. RT-qPCR was performed for the relative quantification of KRT5, KRT20, ESR1, ERBB2 and Calmodulin 2 (CALM2) expression by using gene-specific TaqMan^®^-based assays as described previously [14,22]. Experiments were run on a Roche Light Cycler LC480 (Roche, Germany) according to the following protocol: 5 min at 50 °C, 20 s at 95 °C followed by 40 cycles of 15 s at 95 °C, and 60 s at 60 °C. Forty amplification cycles were applied and the cycle quantification threshold (Ct) values of the gene of interest and the reference gene (CALM2) for each sample (S) were estimated as the median of the triplicate measurements. These were then normalized against the mean expression levels of the REF gene by using the 40-ΔCT method to ensure that normalized gene expression obtained by the test is proportional to the corresponding mRNA expression levels: 40-ΔCT(GOI)S = 40-(CT[GOI]S − CT[REF]S).

### 2.4. Statistical Analyses

Medians and interquartile ranges (IQR) were generated for continuous variables and frequencies and proportions for categorical variables. Afterwards, nonparametric testing, the student’s *t*-test and chi^2^ test were conducted to examine differences in continuous and categorical variables as appropriate. Correlation analyses were performed using Spearman rank correlations. Finally, partition models were generated to create contingency tables with objective cut-offs. All *p*-values were two-sided and statistical significance was assumed at *p* < 0.05. Statistical analyses were performed by JMP SAS 14.3 (SAS, Cary, NC, USA) and GraphPad Prism 7.2 (GraphPad Software Inc., La Jolla, CA, USA).

## 3. Results

### 3.1. Patient and Treatment Characteristics

We included 54 MIBC patients that underwent transurethral resection with consecutive RC after NAC in the final study cohort (Figure 1). The baseline characteristics of these patients stratified by gender are shown in Table 1. There were no statistically significant differences between the two groups in terms of age, BMI, or primary MIBC diagnosis. Treatment characteristics are shown in Table 2.

### 3.2. Elevated Expression of Markers Associated with Luminal Differentiation Favours pCR after NAC

Tumours were considered as luminal type if they had a high expression of ERBB2, ESR1, or KRT20 while showing a low expression of KRT5. With this classification, 35 (64.8%) were regarded as luminal and the other 19 (35.2%) as basal type. Luminal differentiation showed a very strong positive association with a pCR after NAC (*p* = 0.004, Table 3).

### 3.3. ERBB2 Expression Correlates with Luminal Marker Expression

Elevated ERBB2 mRNA expression correlated positively (Spearman’s ρ = 0.56, *p* < 0.0001) with the expression of the luminal marker KRT20 and negatively (ρ = −0.32, *p* = 0.02) with the expression of the basal marker KRT5. KRT20 and KRT5 showed significantly inverse expression (ρ = −0.32, *p* = 0.02). In contrast, ESR1 mRNA expression did not show any significant correlation with either basal or luminal expression patterns (KRT5: ρ = −0.02, *p* = 0.9; KRT20: ρ = −0.03, *p* = 0.83, Table 4).

### 3.4. Comparison of Gene Expression Pre- vs. Post Therapy Reveals Dramatic Downregulation of Luminal Markers KRT20 and ERBB2

**Figure 2:**
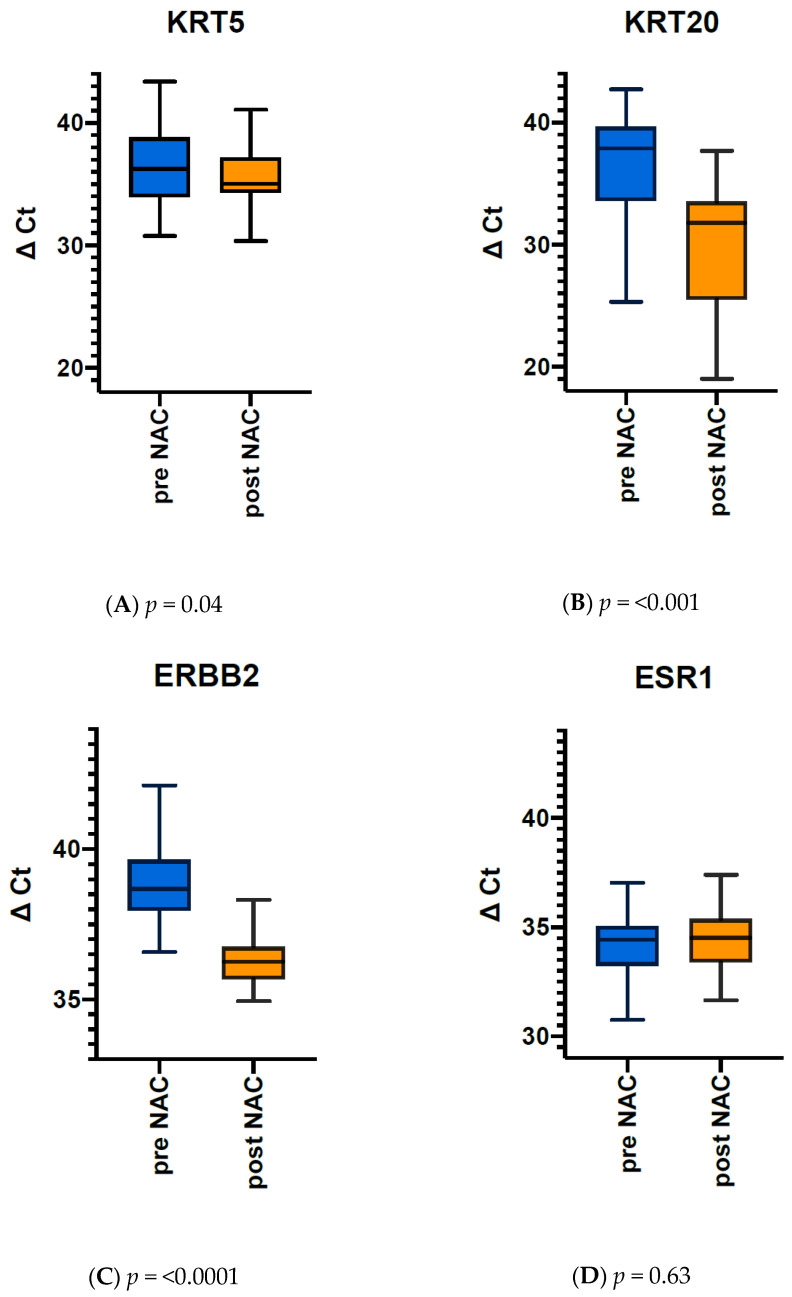
(**A**) KRT5, (**B**) KRT20, (**C**) ERBB2 and (**D**) ESR1 mRNA expression (ΔCT) in MIBC patients before (blue) and after neoadjuvant chemotherapy (post NAC in orange). KRT20 expression decreases significantly after NAC.

Quantitation of KRT5, KRT20, ERBB2, and ESR1 mRNA expression as determined in TUR biopsies before NAC and in cystectomy tissue after chemotherapy revealed substantial dynamic changes (Figure 2). The median KRT5 mRNA expression decreased from 36.17 to 34.9, which reflects a 2fold reduction of KRT5 expression given the logarithmic scale of the normalized gene expression levels (*p* = 0.04, Wilcoxon rank test). Strikingly, the median KRT20 mRNA expression dropped from 37.88 to 31.79, meaning an almost 100-fold downregulation of the luminal marker gene expression (*p* = <0.001). Moreover, 24% of the tumours completely lost KRT20 mRNA expression below the limit of detection. The median ERBB2 mRNA expression also decreased from 38.69 to 36.26 (*p* < 0.0001). In contrast, the median ESR1 mRNA expression did not change significantly before and after chemotherapy (34.44 vs. 34.49 pre- and post-NAC, respectively).

### 3.5. Elevated KRT20, ERBB2 and ESR1 Expression before NAC Correlate with pCR in MIBC Tumours

**Figure 3:**
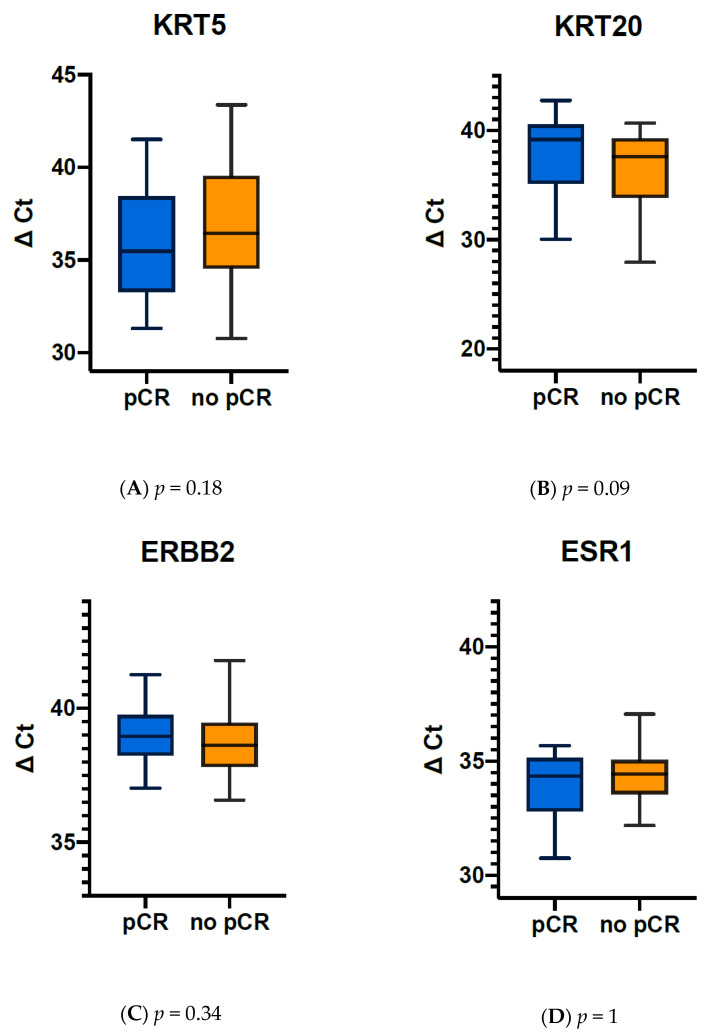
Cytokeratin mRNA expression (ΔCT) in MIBC patients with and without pathological complete response (pCR). (**A**) KRT5 expression, (**B**) KRT20 expression, (**C**) ERBB2 expression, (**D**) ESR1 expression.

The mean expression of KRT20 in MIBC tumours of patients with pCR was 37.09 ΔCt (IQR 5.39 ΔCt, median: 39.15 ΔCt (min: 25.32 ΔCt, max: 42.74 ΔCt)), that in tumours without pCR was 35.6 ΔCt (IQR: 7.05 ΔCt, median: 37.58 ΔCt (min: 19 ΔCt, max: 40.68 ΔCt), Figure 3). The mean expression of ESR1 in tumours showing pCR after NAC was 34.13 ΔCt (IQR: 2.32 ΔCt, median: 34.36 ΔCt (min: 30.77 ΔCt, max: 40.01 ΔCt)), tumours without pCR had a mean expression of ESR1 of 33.81 ΔCt (IQR: 1.58 ΔCt, median: 34.44 ΔCt (min: 19 ΔCt, max: 37.05 ΔCt)).

As the overall expression across all tumours and subtypes revealed differences for KRT20, ERBB2, and ESR1 mRNA expression, the potential predictive value of the target gene expression of KRT20, ERBB2, and ESR1 was further analysed by partitioning test. Interestingly the optimal split for ERBB2 mRNA expression was almost identical to the median expression (38.84 vs. 38.68) and divided the cohort into equally sized groups. For KRT20 the optimal cut off was at 40.485, which is close to the upper quartile of KRT20 mRNA expression at 39.66. Partitioning by ESR1 revealed a best cut off at 34.485, again close to the median expression of 34.09, which is almost identical to the cut off routinely used in breast cancer diagnostics.

Thereafter, the predictive value of ERBB2, ESR1 and KRT20 mRNA expression values were tested by contingency analysis. High expression of the luminal marker KRT20 was associated with a high pCR rate (85.7%), while lower expression of KRT20 in pre-treatment TUR biopsies was associated with lower pCR rate (14.3%, *p* = 0.09, Table 3B).

### 3.6. Changes after NAC in ERBB2 and KRT20 Expression Correlate with Each Other

Changes in ERBB2 correlated positively with changes in KRT20 (Spearman’s with ρ = 0.34, *p* = 0.0133, Table 5). No other significant correlation in changes considering the present gene panel has been observed.

### 3.7. Subanalyses According to Gender

Moreover, potential gender effects were analysed by comparing expression levels of ERBB2 and ESR1 in male vs. female patients. While men were significantly more likely to have pCR (21 male versus 1 female case; *p* = 0.005), there were no significant differences in pretherapeutic ERBB2, ESR1, KRT20, or KRT5 expression according to gender.

## 4. Discussion

The classification of tumour subtypes solely based on histopathological scores may limit the accuracy of staging and subsequent therapeutic management. Molecular profiling with assessment of mRNA from tumour tissues and determination of biomarker expression patterns can contribute to the individualised categorisation of cancer patients and could allow for the prediction of treatment efficacy and outcome parameters.

In the present study, we aimed to determine the clinical value of mRNA expression analysis in tumours from a cohort of 54 MIBC patients who underwent NAC followed by RC. The primary outcome was the association between the expression of the biomarkers KRT20, KRT5, ERBB2, and ESR1 at TUR-BT with pCR at RC. 

Our results demonstrate that urothelial carcinomas with high expression of markers associated with the luminal subtype respond well to platinum-based NAC. Twenty-two of the 54 MIBC patients had pCR after NAC and RC. Elevated expression of ERBB2, KRT20, or ESR1 was found to be indicative of pCR, as 86.4% of pCR patients showed this expression pattern compared to 50% of those without pCR. Peyton et al. demonstrated that pCR is associated with improved overall survival after NAC with consecutive RC [4]. In a recent study, Kriegmair et al. [23] demonstrated that an elevated ERBB2 expression in MIBC tumours was associated with a poor cancer-specific survival in a RC cohort without perioperative chemotherapy. Thus, our findings might indicate that the administration of NAC ameliorates the prognosis of patients with elevated ERBB2 expression. Breyer et al. found a significant correlation between high ERBB2 mRNA expression in tumour tissue from patients with non-muscle-invasive bladder cancer and lower progression-free survival [12]. In the present study, high ERBB2 expression correlated significantly with an elevated KRT20 expression indicative of luminal tumours and with a lower KRT5 expression as a marker for basal tumours. This observation agrees with the notion that ERBB2 is considered a luminal marker in both breast cancers and MIBCs [23]. A drastic decrease of KRT20 expression after NAC could be observed, suggesting that luminal tumours respond exceptionally well to NAC. This confirms the findings of Choi et al. who reported slightly superior response to NAC in their luminal subgroup [13]. In a recent publication, Taber et al. also showed that a high KRT20 expression favours a good response to platin-based NAC [24]. However, the present results based on homogenous cohort of consecutive patients contradict Seiler and colleagues who suggested the greatest benefit of NAC in the subgroup of basal tumours [17]. Both studies were conducted before the consensus classification of molecular subtypes in MIBC was established by Kamoun et al., making a direct comparison of the studies with different categorisations of molecular subtypes difficult [25]. 

Moreover, higher pCR in patients with elevated expression of ERBB2, KRT20 or ESR1 may support previous studies showing a lower cancer-specific [23] and relapse-free [22] survival of MIBC patients with high ESR1-expressing tumours. Importantly, our observed correlation between ERBB2 and ESR1 expression patterns and pCR resembles that shown for breast cancer tissues from patients receiving NAC [15,26] with very similar cut-offs. Denkert et al. demonstrated that 45–50% of breast cancer patients with a pCR in two different cohorts had tumours with high expression levels of ERBB2 [16]. Noske et al. found ERBB2 mRNA expression to be a predictor for a pathological complete response after NAC [26]. These similarities indicate that subtyping of MIBC tumours based on mRNA analysis of ERBB2 and ESR1 expression has a clinical value in the prediction of pCR following NAC, as it is observed for breast cancer patients. It is known from breast cancer that ERBB2 overexpressing tumours respond well to chemotherapy, especially in combination with a taxane-based agent and an anti HER2-antibody, currently being the established neoadjuvant chemotherapy regime [16,27]. To date, the efficacy of this therapy regimen in ERBB2 overexpressing urothelial bladder cancer is unclear [28,29]. To our knowledge, it has not been tested in a neoadjuvant therapeutic intention. It would be interesting to analyse whether pCR rate can be increased if anti HER2-antibody combination therapy is administered in this patient population. Tumours with high expression of both ERBB2 and ESR1 could be of special interest, since these seem to interact with each other under endocrine therapy, providing a resistance mechanism [30,31].

Similar to breast cancer staging, the combination of histopathological evaluation of tumour tissues with molecular biology techniques such as PCR is becoming increasingly important in the appropriate categorisation of MIBC subtypes. Histopathological assessment allows for the visualisation of tissue quality and cellular distribution, yet it is inherently subjective, as it depends on the judgement of the observer and can render semi-quantitative results. In contrast, RNA expression analysis provides quantitative and objective results under standardised conditions. The assessment of biomarker expression levels should supplement the immunohistochemical evaluation of tumour tissues to improve staging of cancer subtypes, particularly of bladder cancers with their known heterogenous molecular profile.

The interpretation of our results is limited by the relatively small cohort size and the retrospective nature of the present study with all its inherent drawbacks. We did not perform a complete molecular subgroup analysis, but only examined KRT5, KRT20, ESR1 and ERBB2 as surrogate markers. However, our method provides a simple and cost-effective way to test muscle-invasive urothelial carcinomas predictively for the response of platinum-based NAC. The predominance of male patients in the study cohort is usual for bladder cancer. Nevertheless, male predominance has to be kept in mind while interpreting hormonal receptor expression patterns. Moreover, our results must be confirmed in a validation cohort.

In conclusion, we demonstrate herein that MIBC subtypes predict pathological response to NAC, indicating strong phenotypic similarities to breast cancer subtypes, including expression patterns of ESR1 and ERBB2. This raises new scopes for potential new treatment algorithms, based on those already established in breast cancer regimens that warrant urgent further elucidation. These findings could prove useful in the prediction of the outcome after a combination therapy with NAC and RC and allow for the selection of patients most suitable for NAC. Prospective validation of these findings has been initiated. 

## Figures and Tables

**Table 1:** Patient characteristics according to gender neoadjuvant chemotherapy (NAC) (*n* = 54).

	Overall	Male	Female	*p*
**n (%)**	54 (100)	42 (77.8)	12 (22.2)	
**Age (median [Interquartile range IQR])**	64.00 [59.00, 69.00]	63.50 [59.00, 69.75]	66.00 [60.25, 69.00]	0.835
**BMI (median [IQR])**	26.49 [24.40, 29.72]	26.93 [24.85, 29.72]	24.59 [23.45, 26.95]	0.100
**Concomitant CIS, n (%)**	3 (5.7)	3 (7.3)	0 (0.0)	0.799
**cT4, n (%)**	4 (7.4)	3 (5.55)	1 (1.85)	1
**ASA-score 3, n (%)**	22 (40.7)	18 (42.9)	4 (33.3)	0.796
**ESR1 Expression (median [IQR])**	34.44 [33.24, 35.04]	34.58 [33.24, 35.04]	33.91 [33.28, 34.97]	0.546
**ERBB2 Expression (median [IQR])**	38.69 [38.05, 39.63]	38.67 [38.21, 39.61]	38.84 [37.72, 39.61]	0.851

**Table 2:** Treatment characteristics according to gender at radical cystectomy (*n* = 54).

	Overall	Male	Female	*p*
**n (%)**	54 (100)	42 (77.8)	12 (22.2)	
**T category**				**0.044**
**ypT0**	24 (44.4)	21 (50.0)	3 (25.0)	
**ypTis**	5 (9.3)	3 (7.1)	2 (16.7)	
**ypT1**	4 (7.4)	3 (7.1)	1 (8.3)	
**ypT2**	11 (20.4)	10 (23.8)	1 (8.3)	
**ypT3**	8 (14.8)	3 (7.1)	5 (41.7)	
**ypT4**	2 (3.7)	2 (4.8)	0 (0.0)	
**N category**				0.125
**pN0**	45 (84.9)	37 (90.2)	8 (66.7)	
**pN1**	2 (3.8)	1 (2.4)	1 (8.3)	
**pN2**	5 (9.4)	2 (4.9)	3 (25.0)	
**pN3**	1 (1.9)	1 (2.4)	0 (0.0)	
**Concomitant CIS**	8 (14.8)	6 (14.3)	2 (16.7)	1
**ypT0**	24 (44.4)	21 (50.0)	3 (25.0)	0.227
**pCR**	22 (40.7)	21 (50.0)	1 (8.3)	**0.024**
**local downstaging (** **≤pT1)**	33 (61.1)	27 (64.3)	6 (50.0)	0.684
**Downstaging (≤pT1pN0)**	30 (55.6)	26 (61.9)	4 (33.3)	0.154
**ESR1 expression (median [IQR])**	34.50 [33.38, 35.29]	34.23 [33.25, 35.00]	35.56 [34.28, 36.03]	**0.015**
**ERBB2 expression (median [IQR])**	36.26 [35.69, 36.72]	36.20 [35.58, 36.72]	36.42 [36.13, 36.69]	0.129

**Table 3:** Contingency tables of combined KRT20, ERBB2 and ESR1 (A), KRT20 (B) and ESR1 (C) cut-offs at transurethral resection before the start of neoadjuvant chemotherapy in relation to pathological complete response (pCR) at cystectomy.

**(A)**			
***KRT20* (≥40.485 ΔCt)** ***ERBB2* (≥38.84 ΔCt)** ***ESR1* (≥34.485 ΔCt)**	***n* (%)**		
	**no pCR**	**pCR**	
**no**	16 (84.2)	3 (15.8)	19 (100)
**yes**	16 (45.7)	19 (54.3)	35 (100)
**total**	32 (59.3)	22 (40.7)	54 (100)
*Fisher’s p = 0.009*			
**(B)**			
***KRT20* ≥ 40.485 ΔCt**	***n*** **(%)**		**Total**
	**no pCR**	**pCR**	
**no**	31 (66)	16 (34)	47 (100
**yes**	1 (14.3)	6 (85.7)	7 (100)
**total**	32 (59.3)	22 (40.7)	54 (100)
*Fisher’s p = 0.014*			
**(C)**			
***ESR1* ≥ 34.485 ΔCt**	***n*** **(%)**		**Total**
	**no pCR**	**pCR**	
**no**	24 (66.7)	12 (33.3)	36 (100)
**yes**	8 (44.4)	10 (55.6)	18 (100)
**total**	32 (59.3)	22 (40.7)	54 (100)
*Fisher’s p = 0.148*			

**Table 4:** Spearman correlation analyses of KRT5, KRT20, ERBB2 and ESR1 mRNA expression before neoadjuvant chemotherapy, significant results are marked with *.

mRNA	mRNA	Spearman ρ	*p*
ERBB2	KRT20	0.5597	<0.0001 *
ERBB2	KRT5	−0.4098	0.0021 *
KRT20	KRT5	−0.3156	0.0201 *
ERBB2	ESR1	0.2476	0.0711
ESR1	KRT20	−0.0305	0.8265
ESR1	KRT5	−0.0180	0.8975

**Table 5:** Spearman correlation analyses of Δ-ΔCT = ΔCT [RC] − ΔCT [before NAC]) KRT5, KRT20, ERBB2 and ESR1; significant results are marked with *.

Δ-ΔCt	Δ-ΔCt	Spearman ρ	*p*
ERBB2	KRT20	0.33501	0.0133 *
ERBB2	KRT5	−0.15109	0.2755
KRT20	KRT5	0.13547	0.3287
ERBB2	ESR1	0.1235	0.3736
KRT20	ESR1	0.2143	0.1197
ESR1	KRT5	−0.0002	0.9989

## Data Availability

No new data were created or analyzed in this study. Data sharing is not applicable to this article.
